# Association between gut microbiota and bone metabolism: Insights from bibliometric analysis

**DOI:** 10.3389/fphys.2023.1156279

**Published:** 2023-04-19

**Authors:** Zhanrong Zhang, Zheng Zhang, Haoming Shu, Yichen Meng, Tao Lin, Jun Ma, Jianquan Zhao, Xuhui Zhou

**Affiliations:** ^1^ Department of Orthopedics, Changzheng Hospital, Second Military Medical University (Naval Medical University), Shanghai, China; ^2^ Department of Orthopedic Rehabilitation, Qingdao Special Servicemen Recuperation Center of PLA Navy, Qingdao, China; ^3^ Department of Orthopedics, Shanghai General Hospital (Shanghai First People’s Hospital), Shanghai Jiao Tong University School of Medicine, Shanghai, China; ^4^ Translational Research Center of Orthopedics, Shanghai General Hospital (Shanghai First People’s Hospital), Shanghai Jiao Tong University School of Medicine, Shanghai, China

**Keywords:** bibliometric, gut microbiota, bone metabolism, hotspots, research trend

## Abstract

Gut microbiota has been reported to participate in bone metabolism. However, no article has quantitatively and qualitatively analyzed this crossing field. The present study aims to analyze the current international research trends and demonstrate possible hotspots in the recent decade through bibliometrics. We screened out 938 articles meeting the standards from 2001 to 2021 in the Web of Science Core Collection database. Bibliometric analyses were performed and visualized using Excel, Citespace, and VOSviewer. Generally, the annual number of published literatures in this field shows an escalating trend. The United States has the largest number of publications, accounting for 30.4% of the total. Michigan State University and Sichuan University have the largest number of publications, while Michigan State University has the highest average number of citations at 60.00. Nutrients published 49 articles, ranking first, while the Journal of Bone and Mineral Research had the highest average number of citations at 13.36. Narayanan Parameswaran from Michigan State University, Roberto Pacifici from Emory University, and Christopher Hernandez from Cornell University were the three professors who made the largest contribution to this field. Frequency analysis showed that inflammation (148), obesity (86), and probiotics (81) are keywords with the highest focus. Moreover, keywords cluster analysis and keywords burst analysis showed that “inflammation”, “obesity”, and “probiotics” were the most researched topics in the field of gut microbiota and bone metabolism. Scientific publications related to gut microbiota and bone metabolism have continuously risen from 2001 to 2021. The underlying mechanism has been widely studied in the past few years, and factors affecting the alterations of the gut microbiota, as well as probiotic treatment, are emerging as new research trends.

## 1 Introduction

Trillions of microorganisms, including bacteria, archaea, fungi, and viruses (Qin et al., 2010), reside in the human gastrointestinal tract (D'Amelio and Sassi, 2018), which constitutes the gut microbiota (GM). In recent years, researchers have come to regard GM as a multicellular organ that influences many physiological functions (Kundu et al., 2017). Inhabiting the surface of the intestinal wall, GM acts as a natural protective film to control nutrient absorption, and GM disturbance is one reason for malnutrition (Mentella et al., 2020). GM may also secrete diverse cytokines that affect the function of insulin, estrogen, and other hormones. This is why GM is now considered an endocrine organ (Qi et al., 2021). Scientists have also found that GM plays a crucial role in the mechanism of metabolism and neurophysiology through the gut-liver-axis and gut-brain-axis (D'Amelio and Sassi, 2018) (Morais et al., 2021). GM has a close relationship with overall health, and any minor imbalances can lead to diseases.

Bone metabolism is a dynamic process that is maintained by the complex network of various types of cells (Lu et al., 2021). Among them, the precise balance between osteoblasts and osteoclasts is of great importance. Any effects on the regulation of osteoblasts or osteoclasts may cause bone loss. Adiponectin can promote bone formation by suppressing osteoclasts transmission and improving the number of osteoblasts (Liu et al., 2021). Inflammation factors such as IL-11, and IL-6 could regulate osteogenesis (Dong et al., 2022). Kevin et al. found that Lgals3-deficient mice enhanced cortical bone mass during aging (Maupin et al., 2018). Disorders of bone metabolism are the cause of many skeletal diseases, such as osteoporosis, osteosarcomas, and rheumatoid arthritis, which place a heavy burden on both patients and society (Khosla and Hofbauer, 2017; Suzuki et al., 2020; Yang et al., 2020).

Accumulating evidence indicates that GM plays a role in regulating bone metabolism through various mechanisms, such as influencing the inflammatory state, endocrine function, calcium or vitamin D absorption, and bone microenvironment (Lu et al., 2021). Over the past decade, the number of studies related to this topic has significantly increased. However, to date, no study has systematically assessed the relationship between GM and bone metabolism in a bibliometric way. Bibliometric analysis examines all publications to determine the distribution, collaborations, general trends, and hotspots in a particular field, providing a comprehensive overview of the structure and development of the field (Tao et al., 2020a; Tao et al., 2020b; Meng et al., 2021). In this study, we employed bibliometric tools to investigate publications related to GM and bone metabolism from 2001 to 2021, focusing on countries, institutions, journals, authors, literature impact, and research keywords, with the aim of presenting an overall picture of the current status of this field.

## 2 Materials and methods

### 2.1 Data extraction and collection

We conducted a literature search related to GM and bone metabolism in the Web of Science Core Collection (WOSCC) database on 27 May 2022. The time scale of our data is from 1 January 2001 to 31 December 2021, and English is required to be the publication language. We used “[(TS=(flora)] OR TS=(microflora) OR TS=(microbio*) AND [(TS=(gut)] OR TS=(intestinal) OR TS=(gastrointestinal) AND [(TS=(bone) OR TS=(bony)]” and “[TS=(flora)] OR TS=(microflora) OR TS=(microbio*) AND [(TS=(gut)] OR TS=(intestinal) OR TS=(gastrointestinal) AND (TS=(osteo*)” as the Boolean operators to identify relevant literature. Original articles and reviews were selected as candidates for bibliometric analysis.

### 2.2 Data analysis

Several bibliometric tools were used in this article to obtain different results. An online analysis platform of bibliometrics (http://bibliometric.com/) was used to quantify the volume of literature in different years, countries, institutions, and journals. The Bibexcel software was used to analyze cooperation relationships between countries or institutions, and the Pajek software was used to visualize the data network. Excel, Citespace (version 5.8 R3), and VOSviewer were used for reference co-citation, author analysis, keyword cluster, and keyword burst analyses.

## 3 Results and discussion

### 3.1 The output of related literature

**FIGURE 1:**
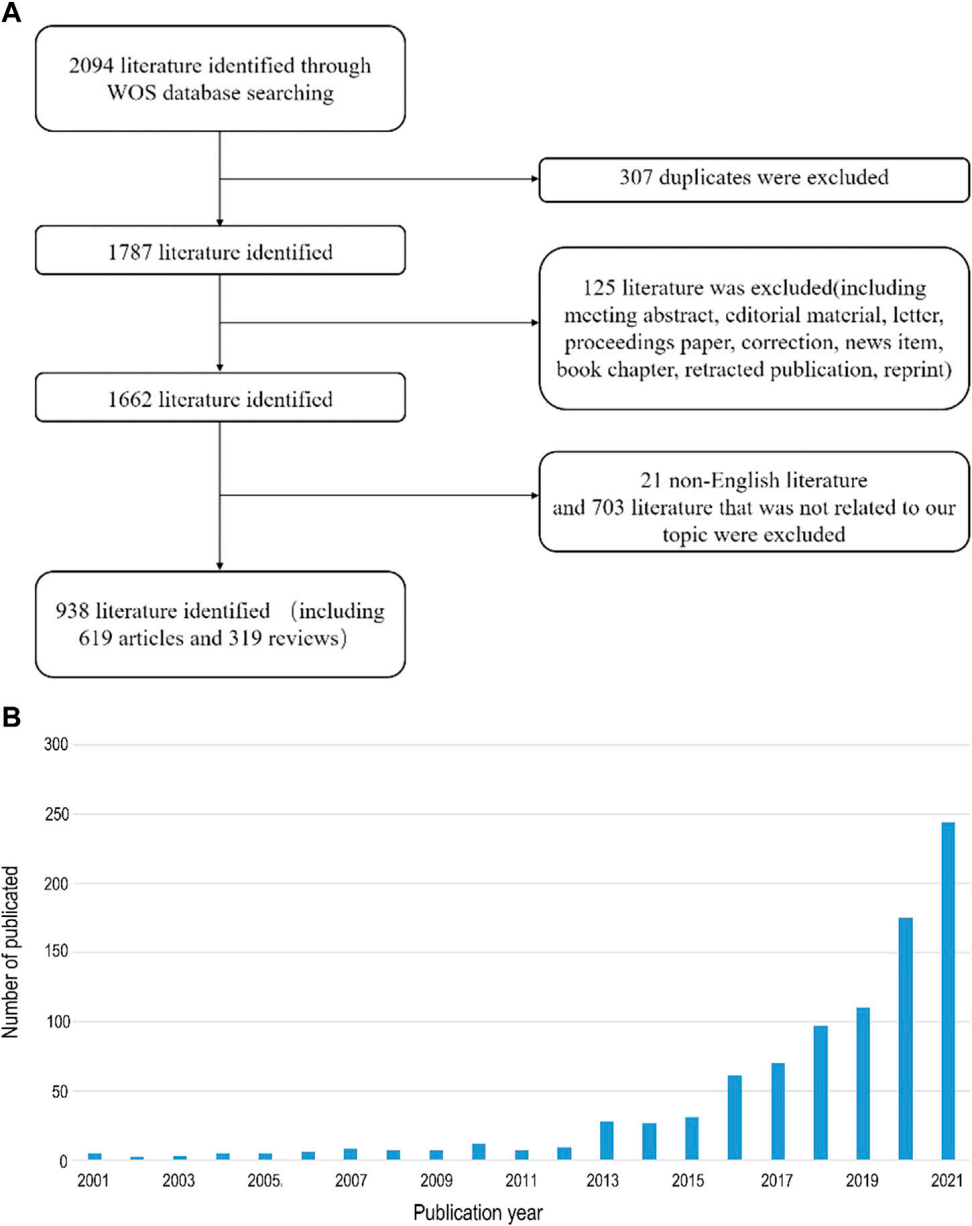
**(A)** Flow chart of screening 938 literature involved in our study. **(B)** Number of publications per year from 2001 to 2021.

To conduct a comprehensive analysis of the literature on the relationship between intestinal flora and bone metabolism, we obtained related articles from WOSCC, the world’s most comprehensive multidisciplinary literature database. Our inclusion criteria resulted in 938 publications (619 original articles and 319 reviews) from 2001 to 2021 (Figure 1A). The number of publications per year is presented in (Figure 1B), indicating a rising trend with slight fluctuations over the past 20 years.

### 3.2 Analysis of publications in different countries and institutions

**FIGURE 2:**
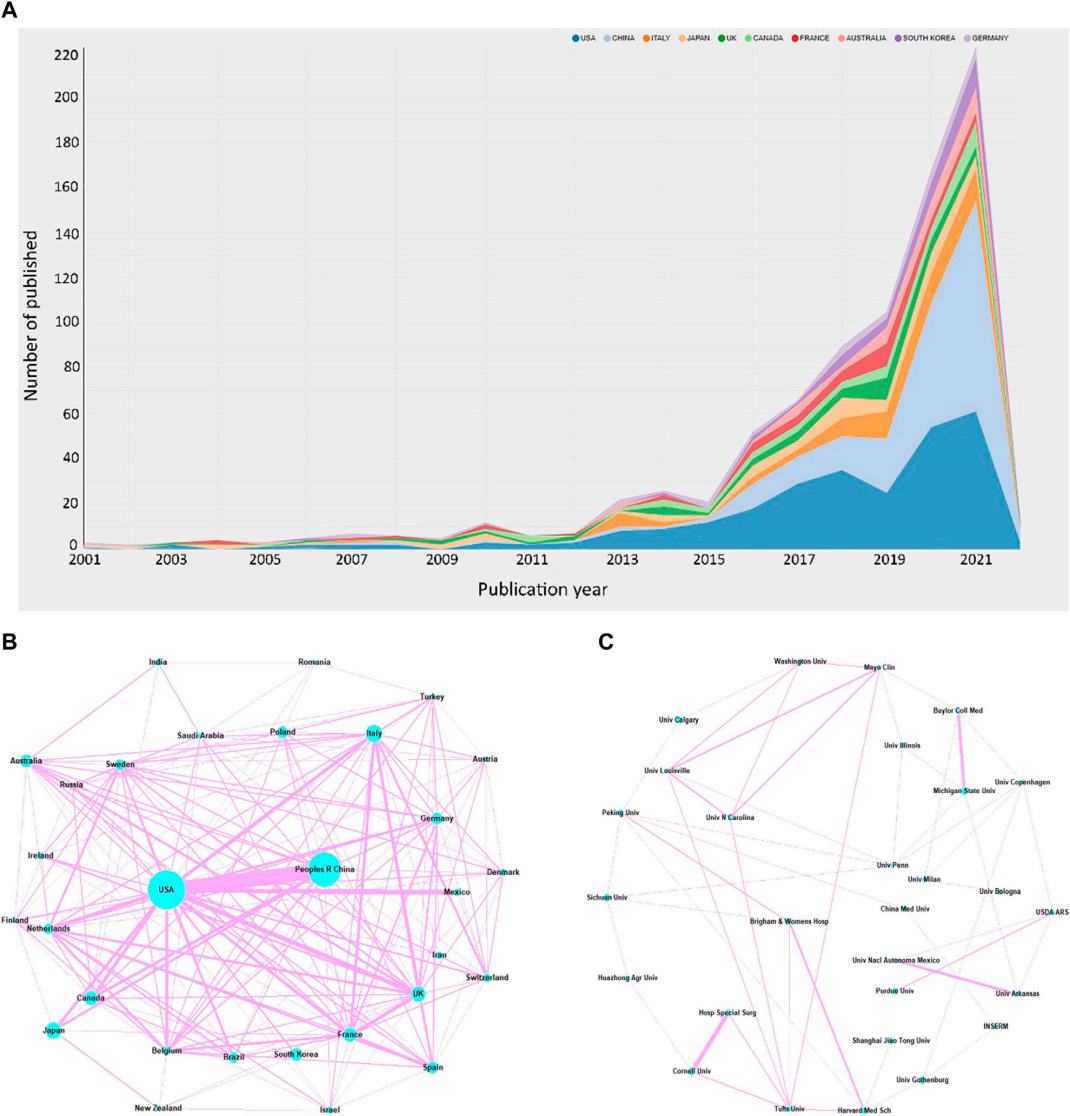
**(A)** Number of publications and increasing trend in the top 10 countries. The network of the cooperation between the top 30 countries **(B)** and top 30 institutions **(C)**.

A total of 73 countries have been involved in this field, with the United States ranking first on the list, having published 283 articles, accounting for 30.4% of the total publications. China and Italy followed closely, ranking second and third, with 220 and 60 publications respectively (Figure 2A). Centrality refers to the influence a country may have on others, with a higher centrality indicating that a country plays a more important role in the cooperation between countries. The United States played a major part in this regard, with a centrality score of 0.24, followed by Italy with 0.14 and France with 0.07 (Table 1A) (Figure 2B).

**TABLE 1A:** The top 10 countries with the highest number of published articles.

Rank	Country	Article counts	Centrality
1	The US	283	0.28
2	China	220	0.06
3	Italy	60	0.14
4	Japan	52	0.01
5	Canada	41	0.03
6	England	41	0.06
7	France	38	0.07
8	Australia	36	0.04
9	South Korea	36	0.00
10	Spain	31	0.02

According to the institution analysis, a total of 1517 institutions conducted studies related to the topic. Among them, Michigan State University and Sichuan University had the highest number of publications (17), followed by the University of Gothenburg (16) and Cornell University (15). Michigan State University, as an American institution, was the most influential because it had the highest number of citations (1020) and the highest average number of citations (60.00) (Table 1B) (Figure 2C).

**TABLE 1B:** The top 10 institutions with the highest number of published articles.

Rank	Institution	Article counts	Total number of citations	Average number of citations	Total number of first author	Total number of first author citations	Average number of first author citations
1	Michigan State Univ	17	1020	60.00	15	394	26.27
2	Sichuan Univ	17	491	28.88	14	80	5.71
3	Univ Gothenburg	16	838	52.38	11	338	30.73
4	Cornell Univ	15	303	20.20	11	151	13.73
5	Emory Univ	14	540	38.57	12	103	8.58
6	Harvard Med Search	13	552	42.46	2	1	0.50
7	Purdue Univ	13	472	36.31	6	110	18.33
8	Shanghai Jiao Tong Univ	11	98	8.91	10	19	1.90
9	Peking Univ	11	214	19.45	7	16	2.29
10	Univ Calgary	11	383	34.82	9	75	8.33

We utilized bibexcel and pajek software to construct a network that provides a more visual representation of the collaboration among countries and institutions. The node size reflects the number of publications, with larger nodes representing a higher number of publications by the country or institution. The line segments connecting the nodes represent the cooperative relationships between them, with the thickness indicating the strength of cooperation. Our analysis revealed that the United States and China had the most frequent cooperation among all countries. The Unites States also had close collaboration with Japan, Canada, the United Kingdom, and Mexico. Additionally, the cooperation tended to occur mostly between countries with high productivity, while less productive countries had fewer publications and opportunities for collaboration. The network of the top 30 most productive institutions showed a low-density state (density = 0.0085), and lacked a central hub connecting most institutions, indicating that no institution had become the authority in this field. Despite having the highest total number of citations, Michigan State University had limited connections with other institutions. Thus, urgent efforts are required to strengthen cooperation between institutions and establish an authoritative center in the field.

Our results indicate that the United States is currently the frontrunner in this field. However, it also suggests that research into the relationship between GM and bone metabolism is still in its early stage.

### 3.3 Analysis of publications in different journals

A journal with a high number of publications signifies that it provides a wide platform for researchers to share their findings, while a journal with a large number of citations indicates that it has significant influence in its field. From 2001 to 2021, 436 journals published articles related to the relationship between GM and bone metabolism. Among these, the top 10 journals published a total of 178 articles, accounting for 18.98% of all publications. Nutrients topped the list with 49 publications, followed by Scientific Reports and International Journal of Molecular Sciences, with 20 and 18 publications, respectively. Interestingly, the journal with the highest number of citations was not the one with the most publications. Journal of Bone and Mineral Research had the largest number of citations (2078), followed by Nutrients (1325) and Journal of Nutrition (1155). In terms of impact factor (IF), Frontiers in Immunology had the highest IF (8.786), while Nutrients (6.706) and Journal of Bone and Mineral Research (6.390) took second and third place, respectively. Six journals belonged to Q1 and four journals belonged to Q2 according to the JCR 2021 classification (Table 2).

**TABLE 2:** The top 10 most active journals with the highest number of published articles.

Rank	Journal title	Article counts	Percentage (N/938, %)	If	Quartile in category	H-index	Total number of citations	Average number of citations
1	Nutrients	49	5.22	6.706	Q1	143	1325	0.94
2	Scientific Reports	20	2.13	4.996	Q2	213	308	3.05
3	International Journal of Molecular Sciences	18	1.92	6.208	Q1	195	297	0.78
4	PLOS ONE	16	1.71	3.752	Q2	367	485	7.81
5	Frontiers in Immunology	15	1.60	8.786	Q1	155	495	0.60
6	Journal of Functional Foods	13	1.39	5.223	Q2	97	89	0.38
7	Calcified Tissue International	12	1.28	4.000	Q2	117	409	9.25
8	Food and Function	12	1.28	6.317	Q1	89	94	0.58
9	Frontiers in Cellular and Infection Microbiology	12	1.28	6.073	Q1	87	43	0.17
10	Journal of Bone and Mineral Research	11	1.17	6.390	Q1	198	2078	13.36

### 3.4 Analysis of the authors contributing to the topic and the most cited literature

(Table 3) lists the top 10 most productive authors in the field, with Narayanan Parameswaran from Michigan State Univ, Roberto Pacifici from Emory Univ, and Christopher Hernandez from Cornell Univ tied for first place with 12 publications each. Meanwhile, Robert Britton from Baylor College had the most citations with 324, but none of these authors had become an authoritative figure in the research on the relationship between gut microbiota and bone metabolism, as their centrality scores were all below 0.1. This suggests that the research in this area is still scattered without a clear focus.

**TABLE 3:** The top 10 most active authors and co-cited authors contributed to the research.

Rank	Author	Article counts	Total number of citations	Average number of citations	Centrality	First author citations counts	Average first author citation counts	Corresponding author citation counts	Co-cited author	Citation counts	Centrality
1	Parameswaran, N	12	312	26.00	0.07	0	0	1	Li, J	272	0.09
2	Pacifici, R	12	139	11.58	0.04	2	11.5	9	Ohlsson, C	196	0.06
3	Hernandez, CJ	12	152	12.67	0.08	4	16	11	Weaver, CM	175	0.05
4	Liu, Y	11	16	1.45	0.04	1	9	0	Cani, PD	173	0.03
5	Maccabe, LR	11	251	22.82	0.08	2	6	3	Sjogren, K	171	0.02
6	Ishimi, Y	11	32	2.91	0.00	2	3	8	Yan, J	165	0.05
7	Weaver, CM	10	154	15.40	0.06	2	29.5	8	Maccabe, LR	159	0.02
8	Chen, J	10	57	2.70	0.05	2	1	1	Setchell, K	145	0.00
9	Ohlsson, C	9	303	33.67	0.08	5	35.2	1	Wang, Y	130	0.00
10	Britton, RA	9	324	36.00	0.03	1	114	3	Britton, RA	126	0.00

Co-cited author analysis can provide insight into the basic research foundation of a specific field, as it highlights the authors whose work has been cited by multiple researchers. Jau-Yi Li was the most cited co-cited author in this field, with 272 citations, followed by Claes Ohlsson (196) and Connie M Weaver (175). However, like the most productive authors, their centrality scores were below 0.1, indicating that the basic research foundation for the relationship between gut microbiota and bone metabolism has yet to be established.

The top 10 most cited articles in this field were concentrated between 2012 and 2018, which reflects the current trend in this area. These highly cited articles can provide insight into the research direction and hotspots in the field. The most cited article was “Gut microbiota induce IGF-1 and promote bone formation and growth” by Julia F. Charles, published in PNAS, which was cited 264 times. The second most cited article was “Sex steroid deficiency–associated bone loss is microbiota dependent and prevented by probiotics” by Roberto Pacifici, published in JCI, which was cited 251 times. Two of the 10 articles were written by the top 10 most productive authors mentioned earlier, and two were written by Klara Sjogren, who was also one of the top 10 co-cited authors (Table 4).

**TABLE 4:** The top 10 highest cited literature related to the research.

Rank	Title	Journal	Corresponding author	Publication year	Total number of citations
1	Gut microbiota induce IGF-1 and promote bone formation and growth	PNAS	Julia F. Charles	2016	264
2	Sex steroid deficiency–associated bone loss is microbiota dependent and prevented by probiotics	JCI	Roberto Pacifici	2016	251
3	The Gut Microbiota Regulates Bone Mass in Mice	JBMR	Klara Sjogren	2012	250
4	Probiotic L. reuteri Treatment Prevents Bone Loss in a Menopausal Ovariectomized Mouse Model	Cellular Physiology	Robert Britton	2014	235
5	Short-chain fatty acids regulate systemic bone mass and protect from pathological bone loss	Nature Communication	Mario M. Zaiss	2018	197
6	Galacto-oligosaccharides increase calcium absorption and gut bifidobacteria in young girls: a double-blind cross-over trial	British Journal of Nutrition	Connie M. Weaver	2018	124
7	Probiotic use decreases intestinal inflammation and increases bone density in healthy male but not female mice	Journal of Cellular Physiology	Robert Britton	2013	120
8	Probiotics Protect Mice from Ovariectomy-Induced Cortical Bone Loss	PLOS ONE	Klara Sjogren	2015	114
9	Microbiota from Obese Mice Regulate Hematopoietic Stem Cell Differentiation by Altering the Bone Niche	Cell Metabolism	Aline Bozec	2015	99
10	Probiotics (Bifidobacterium longum) Increase Bone Mass Density and Upregulate Sparc and Bmp-2 Genes in Rats with Bone Loss Resulting from Ovariectomy	BioMed Research International	Rosita Jamaluddin	2015	94

### 3.5 Analysis of the keywords of the topic

Keyword analysis is a crucial step in identifying research hotspots and predicting future directions for new researchers. In this study, we evaluated keywords in four aspects, with the aim of providing a comprehensive picture of research trends.

**FIGURE 3:**
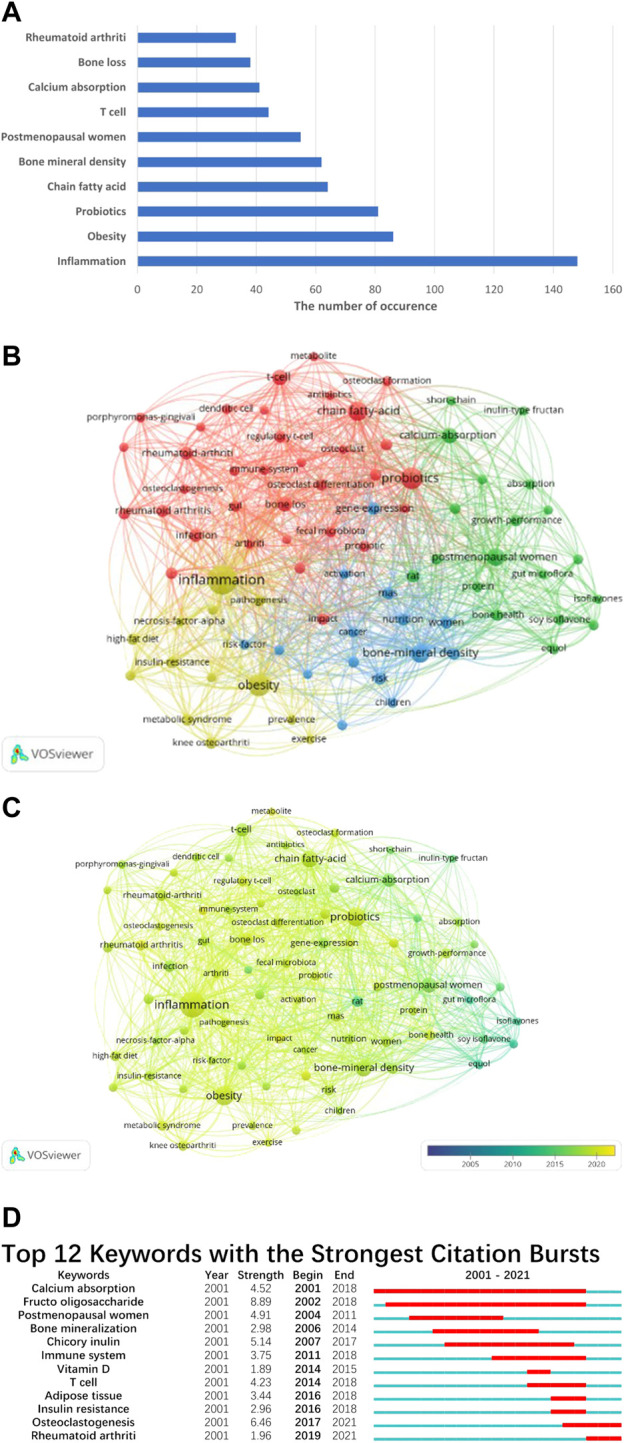
**(A)** The top 10 keywords with the highest occurrences. All keywords are classified into 4 clusters **(B)**, and we also analyze the average appearance time of each keyword **(C)**. The color of the node turning to be bluer means it appears earlier, and more yellow of the node means it appears later. **(D)** The top 12 keywords with the highest burst strength. The blue bar indicates when the keyword appears, and the red bar indicates when the keyword becomes a hotspot.

We have tallied the frequency of keyword occurrences and identified the top 10 keywords for analysis (Figure 3A). The keywords with the highest occurrence rates were inflammation (148), obesity (86), probiotics (81), fatty acids (64), bone mineral density (62), postmenopausal women (55), T cells (44), calcium absorption (41), bone loss (38), and rheumatoid arthritis (33).

We utilized VOS viewer software to classify the keywords into four distinct clusters, as depicted in (Figure 3B). Each cluster is denoted by a unique color. Cluster 1, represented by probiotics, fatty acids, and osteoclastogenesis, primarily focuses on novel preventive approaches and therapeutic targets. Cluster 2, predominantly represented by postmenopausal women, calcium absorption, and growth performance, emphasizes the pathogenesis of bone loss. Cluster 3, represented by inflammation, obesity, and metabolic syndrome, highlights the risk factors that may contribute to the imbalance of gut microbiota. Finally, cluster 4, characterized by bone mineral density, women, children, and nutrition, primarily focuses on the risk factors associated with bone loss.

We also calculated the average appearance time of these keywords (Figure 3C). The research on GM and bone metabolism began with equol and soy isoflavones. However, in recent years, keywords such as rheumatoid arthritis (average time: 2019.61), metabolite (average time: 2019.53), inflammation (average time: 2019.09), probiotics (average time: 2019.02), and obesity (average time: 2018.91) have dominated the field. Burst analysis of keywords not only identifies the prominent keywords during a specific period but also indicates emerging research trends. In our study, “Fructo oligosaccharide” had the highest burst strength (8.89), followed by “Osteoclastogenesis” (6.46) and “Chicory inulin” (5.14). All 12 keywords covered the period from 2001 to 2021. “Calcium absorption” had the longest duration, from 2001 to 2018, followed by “Fructo oligosaccharide,” which lasted for 16 years, from 2002 to 2018 (Figure 3D). Based on the above results, “inflammation,” “obesity,” and “probiotic” were repeatedly mentioned, which caught our attention, and we would like to discuss these three points further.

Previous studies have demonstrated that disturbances in GM can lead to various health issues such as diabetes, obesity, inflammatory bowel disease, rheumatoid arthritis, and colorectal cancer (Illiano et al., 2020; Wu et al., 2020) (Wong and Yu, 2019). Since the first researcher proposed the idea of a relationship between GM and bone metabolism in 2001, numerous studies have been conducted to elucidate the underlying mechanisms. Scientists have identified several possible links between these two research areas, with the most important being the modulation of immunity by the microbiota (Zaiss et al., 2019). This effect on the immune system is a crucial factor in pathological bone loss (Schluter et al., 2020). The critical cytokines associated with bone loss are tumor necrosis factor (TNF), interleukin 6 (IL-6), and receptor activator of nuclear factor-kB ligand (RANKL). On one hand, the GM acts as a protective barrier against foreign pathogens. On the other hand, the GM itself can be considered a foreign pathogen (Jandhyala et al., 2015). Disruption of the GM can trigger an immune response locally or throughout the body, leading to abnormal amounts of molecules related to bone loss. TNF-α, for example, can increase the concentration of c-fms and activate the RANKL pathway, which is a crucial mechanism for osteoclast formation and bone absorption. TNF-α also suppresses the release of osteoprotegerin, which alleviates the inhibition of osteoclast activity (Yao et al., 2021). The effect of IL-6 on osteoclasts is similar to that of TNF-α, and it can also enhance osteoclast activity and promote bone loss (Zhou et al., 2019). Moreover, Luo et al. found that *Clostridium* can promote the accumulation of regulatory T cells (Treg), which inhibit osteoclast differentiation, in the colonic lamina propria (Luo et al., 2011). Schepper et al. found that *Lactobacillus* reuteri can reduce the number of T lymphocytes and inhibit the formation of osteoclasts (Schepper et al., 2020).

Obesity was previously thought to be a protective factor against bone loss due to the higher bone mineral rate found in obese bodies (Villareal et al., 2005). However, conflicting views on the impact of obesity on bone health have emerged with studies on bone microarchitecture, and the latest evidence suggests that obesity is detrimental to bone metabolism (Rinonapoli et al., 2021). One possible reason for this is that obesity is associated with systemic inflammation, which negatively affects bone health, especially in individuals with abdominal obesity (Qiao et al., 2021). Adipose tissue, which has endocrine functions, may also contribute to this negative effect by increasing the level of leptin and suppressing the level of adiponectin, both of which have negative effects on bone health (Yue et al., 2016). Additionally, obesity is linked to a range of complications that affect bone metabolism, such as the differentiation of bone mesenchymal stem cells (BMSC) (Wang et al., 2021). Obesity is also closely related to GM, as GM disorder is a major cause of obesity (Cuevas-Sierra et al., 2019). This disorder can lead to changes in intestinal absorption function, as microbiota in obese phenotyped mice may consume more carbohydrates and protein for energy (Dabke et al., 2019). GM also releases various hormones, including 5-Hydroxytryptamine (5-HT) and lactate, to regulate central appetite (Wu et al., 2019). Moreover, GM metabolites can pass through the intestinal epithelial barrier, and GM disorder can disrupt its function, leading to chronic inflammation and obesity (Tilg et al., 2020). The link between GM and obesity, and the link between obesity and bone metabolism, has led scientists to explore the potential of regulating GM to control obesity as a means of treating osteoporosis. However, there is still much research to be done in this area.

Probiotics and prebiotics have already been used in clinical practice to rebalance GM in the host (Azad et al., 2018; Sorrenti et al., 2020). Probiotics are defined as live microorganisms that provide health benefits to the human body when consumed in sufficient amounts. Prebiotics are non-digestible food components that stimulate the growth of beneficial microorganisms and enhance the activity of the beneficial microbiome (Sanders et al., 2019). Both probiotics and prebiotics have been shown to improve calcium and vitamin D absorption, leading to positive effects on bone formation (Raveschot et al., 2020) (Yoon and Michels, 2021) (Locantore et al., 2020). The first articles reporting the positive effects of probiotics on calcium absorption date back to 2003, and the keyword analysis in our study reveals that fructo-oligosaccharides, a type of prebiotic, first appeared in 2002. With advances in research technology, differences in the gut microbiome between individuals with bone loss and those with healthy bones have become increasingly detailed. Pan et al. (2019). Conducted a metagenomic sequencing study on bone restoration and bacteria and found that the composition of the gut microbiome, such as *Lactobacillus* casei and *Lactobacillus* reuteri, differed significantly between individuals with bone loss and those with healthy bones. Probiotics and prebiotics have also been used in the treatment of other systemic diseases, such as inflammatory bowel disease and Alzheimer’s disease (Jakubczyk et al., 2020) (Kesika et al., 2021). The use of probiotics or prebiotics to restore bone formation has also been shown to be effective. For example, Zhang et al. demonstrated that the administration of fructo- and galacto-oligosaccharides can effectively protect bones from osteopenia in obese individuals (Zhang et al., 2021). Behera et al. found that probiotics and their metabolites can influence bone metabolism *via* the gut-bone axis (Behera et al., 2021). However, identifying the most effective probiotics or prebiotics, or combinations of these ingredients, remains an area of unexplored research and is likely to be a major research focus in the future.

Still, there are certain limitations in our research. The studies included in our research are from 2001 to 2021, without the articles published in 2022. Due to the timely update of the WOSCC database and the emergence of new articles, the real-time result of bone metabolism and GM bibliometric analysis may have some deviations. Nevertheless, these deviations are not very significant, which will not affect the research trend and conclusion we get in our analysis. Our results are still reliable and creditable.

## 4 Conclusion

In this article, we have conducted a bibliometric analysis of articles related to bone metabolism and GM published from 2001 to 2021. Our research highlights the crucial role of GM in affecting bone metabolism, which has garnered significant interest from researchers worldwide. Notably, studies have focused not only on elucidating the underlying mechanisms but also on developing interventions to alleviate osteoporosis by modulating GM.

Over the past two decades, there has been a steady increase in research on the relationship between GM and bone metabolism, and this trend shows no signs of slowing down. Studies have identified inflammation as the most critical underlying mechanism linking GM and bone metabolism. New research trends have emerged, such as investigating interventions to prevent obesity-related bone loss and developing probiotics to restore GM balance, which have become the new hotspots in this field.

We believe that further research aimed at elucidating the underlying mechanisms linking GM and bone metabolism will advance our understanding of osteoporosis therapy. Such research will contribute to the development of novel interventions that can modulate GM to promote bone health and prevent osteoporosis. Therefore, we urge continued investment in this field to uncover new insights and develop effective treatments for this debilitating condition.
